# The relative length of the patient and the primary care interval in patients with 28 common and rarer cancers

**DOI:** 10.1038/bjc.2015.40

**Published:** 2015-03-03

**Authors:** G Lyratzopoulos, C L Saunders, G A Abel, S McPhail, R D Neal, J Wardle, G P Rubin

**Affiliations:** 1 Health Behaviour Research Centre, Department of Epidemiology and Public Health, University College London, 1-19 Torrington Place, London WC1E 6BT, UK; 2 Cambridge Centre for Health Services Research, Department of Public Health and Primary Care, University of Cambridge, Cambridge CB2 0SR, UK; 3 National Cancer Intelligence Network (NCIN), Public Health England, 5th Floor, Wellington House, 135-155 Waterloo Road, London SE1 8UG, UK; 4 North Wales Centre for Primary Care Research, College of Health and Behavioural Sciences, Bangor University, Gwenfro Unit 5, Wrexham Technology Park, Wrexham LL13 7YP, UK; 5 Wolfson Research Institute, School of Medicine and Health, University of Durham, Queen's Campus, University Boulevard, Stockton-on-Tees TS17 6BH, UK

**Keywords:** patient, primary care, interval, public, health, interventions, symptoms

## Abstract

**Background:**

Appreciating variation in the length of pre- or post-presentation diagnostic intervals can help prioritise early diagnosis interventions with either a community or a primary care focus.

**Methods:**

We analysed data from the first English National Audit of Cancer Diagnosis in Primary Care on 10 953 patients with any of 28 cancers. We calculated summary statistics for the length of the patient and the primary care interval and their ratio, by cancer site.

**Results:**

Interval lengths varied greatly by cancer. Laryngeal and oropharyngeal cancers had the longest median patient intervals, whereas renal and bladder cancer had the shortest (34.5 and 30 compared with 3 and 2 days, respectively). Multiple myeloma and gallbladder cancer had the longest median primary care intervals, and melanoma and breast cancer had the shortest (20.5 and 20 compared with 0 and 0 days, respectively). Mean patient intervals were longer than primary care intervals for most (18 of 28) cancers, and notably so (two- to five-fold greater) for 10 cancers (breast, melanoma, testicular, vulval, cervical, endometrial, oropharyngeal, laryngeal, ovarian and thyroid).

**Conclusions:**

The findings support the continuing development and evaluation of public health interventions aimed at shortening patient intervals, particularly for cancers with long patient interval and/or high patient interval over primary care interval ratio.

Most patients with cancer present with symptoms, and of those most first present to a general practitioner (Elliss-Brookes *et al*, 2012; Jensen *et al*, 2014). For these patients, timeliness of diagnosis is a function of both the length of time from symptom onset to first consultation (the patient interval), and the time from first consultation to specialist referral (the primary care interval; Weller *et al*, 2012). Interventions aiming at shortening either interval have been developed, with either a predominantly community focus (e.g., public health interventions to help individuals recognise, and act on, ‘alarm symptoms') or a health-care system focus (e.g., decision-support interventions or specialist referral guidelines; NICE, 2005; Austoker *et al*, 2009; Hamilton *et al*, 2013).

The relative contribution of each of the two intervals (i.e., the patient or the primary care interval) to the length of the overall pre-referral interval from symptom onset to referral is nonetheless inadequately described for most cancers. The majority of the evidence relates to methodologically heterogeneous studies examining either the patient or the primary care interval on their own, often focussing on a single or a few cancer sites. However, some recent studies have encompassed multiple cancer sites, identifying large differences between cancers in the length of various diagnostic intervals (Baughan *et al*, 2009; Tokuda *et al*, 2009; Hansen *et al*, 2011; Lyratzopoulos *et al*, 2013a; Keeble *et al*, 2014; Neal *et al*, 2014). In addition, current evidence suggests that primary care intervals tend to be very short for cancers in which most patients present with palpable or visible symptoms and signs, such as for breast cancer, melanoma and endometrial cancer (Baughan *et al*, 2009; Lyratzopoulos *et al*, 2013a), which also tend to be associated with socioeconomic inequalities in stage at diagnosis (Lyratzopoulos *et al*, 2013b; Robbins *et al*, 2014). These observations indicate that patient intervals may dominate the length of overall pre-referral intervals for at least some cancers. In contrast, the relative contribution of the primary care interval may be greater for other cancers, particularly those in which substantial proportions of patients present with symptoms of low specificity and which are considered ‘harder to suspect' after presentation (Lyratzopoulos *et al*, 2012). In order to better target interventions aiming to shorten the pre-referral interval, the relative contribution of pre- and post-presentation intervals for different cancers needs to be better described.

We therefore set out to comprehensively examine differences in the length of patient and primary care intervals for a range of common and rarer cancers. In doing so, we systematically address variation by cancer in the length of the two intervals to help inform decision-making about the development, implementation and evaluation of either community- or primary care-based interventions.

## Patients and methods

We analysed data from the (English) National Audit of Cancer Diagnosis in Primary Care 2009–2010. Details of the methods used in the audit have been published previously (Rubin *et al*, 2011). Briefly, using continuous sampling during the audit period, data on aspects of the diagnostic process of cancer patients were collected by general practitioners or other primary care professionals in an estimated total of 1170 general practices (∼14% of all practices in England) using information included in practice patient records. Data collection excluded screening-detected cancers and non-melanoma skin cancer. Although practices participated voluntarily, comparisons with cancer registration statistics indicate that the data set is representative of the age, sex and cancer site case-mix of incident English cancer patients (Rubin *et al*, 2011). Further, care quality measures (Quality and Outcomes Framework and General Practice Patient Survey practice scores) and other practice characteristics were similar in participating and non-participating practices belonging to the same (former) cancer networks (Lyratzopoulos *et al*, 2013c).

### Interval measures

The patient interval was defined as the period between symptom onset and first relevant presentation, defined as the first notification to any health-care professional working within the primary care team about a symptom or sign that was probably due to the cancer, based on information available in the medical records (Rubin *et al*, 2011; Weller *et al*, 2012). The primary care interval was defined as the period from the first relevant symptomatic presentation to a general practitioner and their first specialist referral for further investigation (Rubin *et al*, 2011; Weller *et al*, 2012). For each patient, we also calculated the overall pre-referral interval (denoting the period from symptom onset to referral) by summing patient and primary care intervals. Information was also available on the referral interval (the period from the date of first consultation to the first hospital appointment for specialist assessment).

### Analysis

The analysis sample included patients aged 15 years or older who had first presented to a general practitioner and were subsequently diagnosed with one of 28 cancers (i.e., bladder, brain, breast, cervical, colorectal, endometrial, gallbladder, laryngeal, leukaemia, liver, lung, lymphoma, melanoma, mesothelioma, myeloma, oesophageal, oropharyngeal, ovarian, pancreatic, prostate, renal, sarcoma, small intestine, stomach, testicular, thyroid, unknown primary or vulval cancer). We calculated the mean, median, 25th, 75th and 90th centiles (and respective 95% confidence intervals (CIs), estimated using a bootstrap approach) for the patient, primary care and overall pre-referral intervals, by cancer site. Further, we calculated the ratio of the mean patient interval to the mean primary care interval for each cancer, and similarly the ratio of respective median values. In supplementary analysis, for each cancer separately, we also present information on the referral interval (date of referral to date of first hospital appointment) and the proportion of patients with interval values 0–14, 0–30 and 0–90 days to enable future comparisons with literature reporting binary proportions of patients exceeding the respective cut-off interval values.

## Results

Of 14 931 patients with any of the 28 cancers being considered, who presented to a general practitioner and were older than 15, 3978 patients (26.6%) had a missing (or invalid) patient or primary care interval value; therefore, analysis was confined to 10 953 patients. The proportion of cases excluded because of missing interval data was <28% for all but five cancers (leukaemia, prostate, liver, melanoma and multiple myeloma for which it was 45%, 44%, 40%, 39% and 34%, respectively). Sample sizes for each cancer site reflected its population incidence, ranging from 38 patients with small intestine and 40 with gallbladder cancer to 1673 patients with colorectal and 2124 with breast cancer.

### Variation in the patient interval

Laryngeal and oropharyngeal cancers had the longest median patient intervals (34.5 (95% CI: 30–57) and 30 (95% CI 21–34) days, respectively), whereas renal and bladder cancer had the shortest (3 (95% CI 1–5) and 2 (95% CI 1–3) days, respectively; Table 1). Another seven cancers (cervical, oesophageal, melanoma, thyroid, colorectal, mesothelioma and vulval cancer) had median patient intervals between 17 and 25 days. In addition to variation in medians, the distribution of the patient interval also varied between cancers. In particular, some cancers with relatively short median patient intervals had relatively long 75th and 90th centile intervals – for example, testicular and small intestine cancer, with medians of 12 and 10.5 days, respectively, but 75th centile values of 67 and 75 days. The opposite pattern was also observed, where the median interval is relatively long compared with other cancers, but the 75th centile is relatively short – e.g., oesophageal cancer.

### Variation in the primary care interval

Multiple myeloma and gallbladder cancer had the longest median primary care intervals (20.5 (95% CI 14–31) and 20 (95% CI 9–29) days, respectively) and melanoma and breast cancer had the shortest (0 (95% CI 0–0) days for both; Table 1). Another eight cancers had median primary care intervals between 10 and 15 days. Similarly with the patient interval, there is variation in the 75th and 90th centiles that is not a direct reflection of the variation in the medians.

### Variation in the overall pre-referral interval

The overall pre-referral interval ranged from 58 (95% CI 46–66) days for laryngeal cancer to 10 (95% CI 9–12) days for breast cancer (Table 1). Because some patients have greater than median values for both the patient and the primary care intervals, the median pre-referral intervals for patients with a given cancer are greater than the sum of the medians of the constituent intervals.

### Relative length of patient and primary care intervals

**Figure 1:**
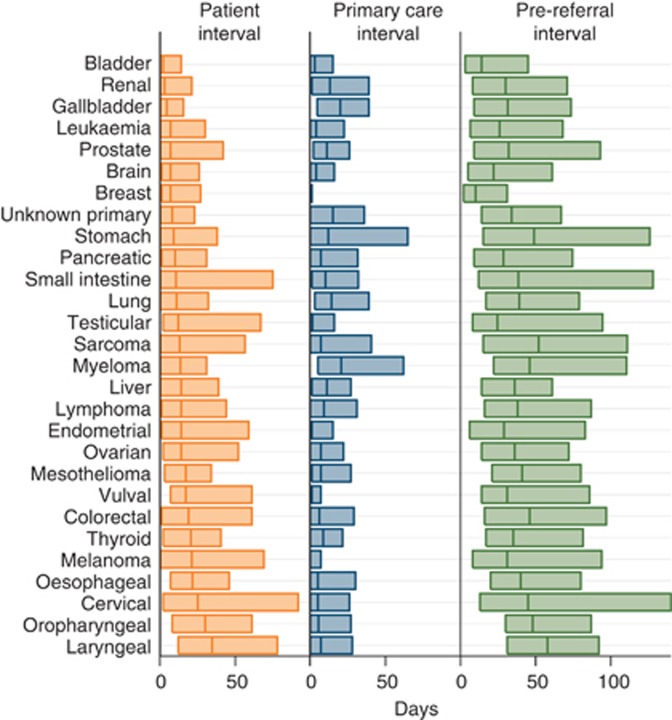
**Visual summary of length of the patient, the primary care and the pre-referral intervals by cancer.** Bar length represents the size of the interquartile interval, with median values depicted by a vertical line. Cancers are ordered in ascending order of median patient interval. Note that median patient intervals tend to be longer than primary care intervals for most cancers, and very short primary care intervals are seen for cancers such as breast, vulval and melanoma.

**Figure 2:**
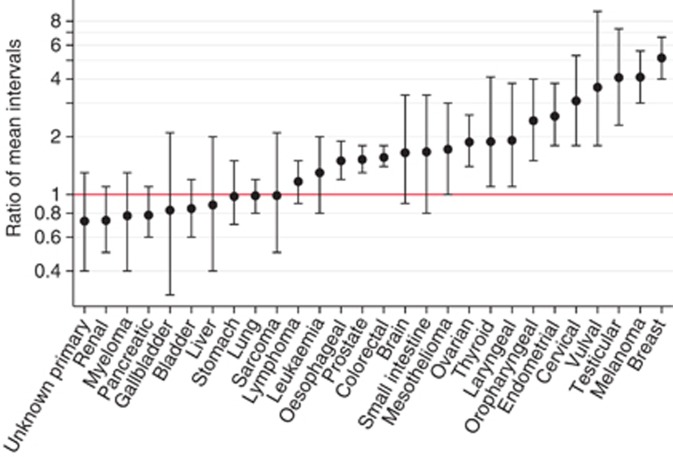
**Visual summary of ratios of means (patient/primary care interval) and related 95% confidence intervals.** Note that the *y* axis is on the logarithmic scale.

Mean and median patient intervals were longer than mean and median primary care intervals for most cancers (18 out of 28 and 20 out of 28, respectively; Table 1). In particular, the mean patient interval was approximately five-fold greater than the mean primary care interval for breast cancer, four-fold greater for melanoma and testicular cancer, three-fold greater for vulval and cervical cancer and two-fold greater for endometrial, oropharyngeal, laryngeal, ovarian and thyroid cancers (Table 2). Figures 1 and 2 pictorially summarise the patient and the primary care interval by cancer site.

### Supplementary analysis

In contrast to the size of variation by cancer in the median length of both the patient and the primary care intervals (i.e., 34.5 *vs* 2 days, and 20.5 *vs* 0 days, respectively), variation by cancer with respect to the referral interval was relatively small, with maximum and minimum median values of 14 days for bladder or thyroid cancer and 5 days for leukaemia (data not shown). Further, the proportions of patients with interval values of 0–14, 0–30 and 0–90 days for the patient, primary care and overall pre-referral intervals are provided in Supplementary Online Material 2.

## Discussion

The length of patient and primary care intervals varies greatly by cancer site. Moreover, the relative contribution of either interval in the overall duration of the pre-referral period is variable, with the average patient interval being at least two-fold longer than the primary care interval for 10 cancers.

### Comparison with other literature

Evidence about the length of patient and primary care intervals in patients with different cancers is sparse, because such measures do not currently form part of population-based cancer registration systems. Therefore, as is the case for the present study, most evidence thus far typically comes from primary care medical record studies (Baughan *et al*, 2009; Tokuda *et al*, 2009; Hansen *et al*, 2011). However, collection of relevant information from primary care records is a time-consuming process, and collation of data from continuous samples is often only possible in the context of national audit initiatives – such as the first English National Audit of Cancer Diagnosis in Primary Care and similar previous initiatives in Scotland and Denmark (Baughan *et al*, 2009; Hansen *et al*, 2011; Rubin *et al*, 2011). The present findings add to prior evidence about diagnostic intervals, previously reported for between 10 and 18 cancers (Baughan *et al*, 2009; Hansen *et al*, 2011; Lyratzopoulos *et al*, 2013a; Keeble *et al*, 2014), by including the largest number of cancer sites examined to date, by additionally describing overall pre-referral intervals, and by comparing the length of patient and primary care intervals for different cancers.

### Strengths and limitations

Strengths of the study include its large sample and the inclusion of patients with 28 common and rarer cancers. Data have been collected by medical or nursing staff on continuous samples of incident cancer cases during the audit period (Rubin *et al*, 2011), thus minimising potential for selection bias. The diagnostic case-mix of included patients was similar to incident cases, and the performance and characteristics of participating general practices were similar to non-participating ones, both these observations indicating a representative sample (Rubin *et al*, 2011; Lyratzopoulos *et al*, 2013c).

There are four principal limitations. First, in contrast to information about the primary care interval (which is obtainable from medical records in a relatively straightforward manner), information about the patient interval requires that patients accurately appreciate and report the duration of their symptoms, and doctors accurately interpret and enter that information into their medical notes. Although information about symptom duration before presentation is nearly always elicited during general practice consultations, inaccuracies in the measurement of the patient interval are likely to occur, particularly in the context of comorbid conditions. However, we assume that, although inaccuracies in the measurement of the patient interval may vary by symptom, they are unlikely to be systematically biased towards either over- or under-estimating its length (Lynch *et al*, 2008). It is also unlikely that inaccuracies in patient recall will be grossly differential between patients with different cancers, given the large overall size of the observed variation (Keeble *et al*, 2014). For example, very substantial levels of differential recall accuracy between patients with bladder and laryngeal cancer would have been required to generate the very large difference in median patient intervals between these two cancers (2 *vs* 34.5 days, respectively).

Second, we were not able to include in the analysis one in four patients in the population of prior interest – chiefly because of missing data about the patient interval (data now shown). Caution is particularly required when interpreting findings for cancers with relatively high proportions of missing interval data – for example, leukaemia and prostate cancer (both with >40% missing interval data). It should be noted that unlike the start of the primary care interval, which is based on the recorded first consultation date, the start of the patient interval was inferred using information in the free text of the patient records. Failure by the GP to note the duration of symptoms would make inferring the start of the patient interval impossible. However, patterns of patient interval variation by cancer are robust to extreme case scenario sensitivity analysis (Keeble *et al*, 2014).

Third, our findings relate to a population of English cancer patients who first attended primary care in the audit period (2009–2010). It is therefore possible that the population values of the reported intervals have changed to some degree since then, given recent interventions to increase public awareness of symptoms or provision of decision-support tools for general practitioners (Hamilton *et al*, 2013; NHS, 2014).

Fourth, although our study sample is relatively large, the exact ordering of different cancers with respect to either interval (and consequently also their ratios) is likely to be subject to considerable uncertainty. Rather than concentrating on the precise ordering of cancers we focus on the overall pattern of variation, focussing on those cancers with the shortest or the longest intervals (and the higher interval ratios).

### Implications

The findings can help inform priorities about interventions aimed at shortening either interval. For six of the cancers with the longest patient interval, large proportions of patients present with fairly typical symptoms – for example, laryngeal (voice hoarseness), oropharyngeal (oral ulcer/lesion), oesophageal (dysphagia), melanoma (pigmented skin lesions) and vulval (vulval ulcer/lesion). The observation that these cancers are associated with long patient intervals despite the presence of clear-cut symptoms in most patients subsequently diagnosed with them would, in principle, advocate the development and evaluation of public health campaigns aimed at raising awareness of symptoms, and appropriate help-seeking behaviour, for these (rarer) cancers. In England, current public health awareness interventions thus far have not as yet encompassed most of the above ‘principal symptom – cancer site' pairs, possibly because of (justifiable) initial emphasis on most common cancers (NHS, 2014), although they could be considered in the future.

The relative contribution of the patient interval in the overall length of the pre-referral interval is also important. Most cancers with two-fold or greater length of patient compared with primary care intervals are characterised by the presence of palpable, visible or noticeable symptoms in most patients – for example, breast (lump), melanoma (pigmented skin lesion), testicular (lump), vulval (vulval ulcer/lesion), endometrial (vaginal bleeding, typically post-menopausal), oropharyngeal (oral ulcer/lesion) and laryngeal (voice hoarseness). These observations advocate the need for either the continuation (where those exist) or the development of new public health interventions aimed at shortening the patient interval associated with those cancers. It should be noted that certain cancers (e.g., laryngeal, oropharyngeal, melanoma and vulval) associated with longest patient intervals also have high patient/primary care interval length ratios.

It should nonetheless be emphasised that, beyond consideration of evidence about the length of patient interval, the selection of symptom – cancer pairs for public health awareness campaigns ought to be informed by a number of factors, including the consideration of the positive predictive value of their common presenting symptoms. Existing campaigns cover symptoms with positive predictive values in the order of 5% (NHS, 2014) – for example, haemoptysis (7.5% and 4.3% for lung cancer in men and women, respectively), haematuria (7.4% and 3.4% for bladder cancer in men and women, respectively), and rectal bleeding (2.4% for colorectal cancer in men) Jones *et al*, 2007. These comparisons provide a pragmatic test about whether other symptom – cancer pairs can be considered as candidates for public health campaigns. Against this background, it should be noted that the positive predictive value of dysphagia for oesophageal cancer in men is 5.7% (Jones *et al*, 2007), whereas that of post-menopausal bleeding for endometrial cancer is 4.0% (Walker *et al*, 2013), that is values that are comparable to those of other symptoms that are already targeted by public health education campaigns. Good-quality evidence about the positive predictive value of oral or vulval ulceration or voice hoarseness in populations of patients consulting in primary care is, however, currently lacking. We strongly support that the selection, design, piloting and implementation of public health awareness campaigns about symptoms likely to be due to cancer require expert multi-disciplinary input (including from epidemiology, psychology and primary care, alongside clinical specialities and other relevant disciplines) and a robust evaluation framework.

On the other hand, several cancers have relatively long primary care intervals, which also form a major part of overall pre-referral intervals (i.e., lung, sarcoma, stomach, gallbladder, myeloma, pancreatic, renal and cancer of unknown primary – these cancers have a patient/primary care interval ratio of 1 or lower). In general, this group is dominated by cancers that have been previously described as ‘harder to suspect' because most patients present with symptoms with particularly low predictive values (Lyratzopoulos *et al*, 2012). For these cancers, continuing efforts to support the diagnostic process after presentation to a general practitioner are needed, including the use of decision-support/risk assessment tools, clinical audit/root cause analysis reviews and widening of access to specialist diagnostics (Lyratzopoulos *et al*, 2014).

Although evidence about the length of the patient and the primary care intervals, and their ratios, can inform the choice and nature of interventions, there may also be opportunities for further targeting such strategies by considering socio-demographic variation in either interval. For example, an ongoing breast symptom awareness campaign is specifically addressing older women (NHS, 2014), as indirect evidence indicates that older women are likely to have longer patient intervals for breast symptoms (Lyratzopoulos and Abel, 2013). Another consideration to be borne in mind is that the median does not capture all facets of variation in intervals between cancers. For example, compared with other cancers, testicular cancer has a typically short median patient interval (of <2 weeks), whereas the upper quartile is longer compared with most other cancers (>2 months). This indicates that most people with testicular cancer consult their GP quickly, but for a substantial minority the interval between symptom recognition and consulting a GP is long.

In conclusion, appreciating variation in the patient and primary care intervals and their relative length can inform priorities for future early diagnosis research and policy strategies, helping to optimally use either a community-based or a health-care system-based focus, or their combination, as applicable for different cancers. Commitment to regular collection of data about both pre-referral diagnostic intervals in representative samples of cancer patients is critical for the evaluation and monitoring of such interventions.

## Figures and Tables

**Table 1:** Summary statistics for the patient, the primary care and the pre-referral intervals expressed in number of days (*n*=10 953 patients with 28 cancers)^a^

		**Patient interval (days)**					**Primary care interval (days)**					**Pre-referral interval (days)**				
**Cancer diagnosis**	**N**	**Mean**	**25th Centile**	**50th Centile**	**75th Centile**	**90th Centile**	**Mean**	**25th Centile**	**50th Centile**	**75th Centile**	**90th Centile**	**Mean**	**25th Centile**	**50th Centile**	**75th Centile**	**90th Centile**
Laryngeal	**94**	68	12	34.5	78	183	36	0	7	28	57	104	31	58	92	234
Oropharyngeal	**146**	53	8	30	61	121	22	0	5.5	27	56	75	30	48	87	136
Cervical	**97**	77	2	25	92	265	25	0	5	26	79	102	13	45	140	293
Oesophageal	**418**	40	7	21.5	46	99	26	0	5	30	71	66	20	40	80	152
Melanoma	**475**	69	0	21	69	234	17	0	0	7	44	86	8	31	94	317
Thyroid	**80**	60	2	20.5	40.5	192.5	32	0	8.5	21.5	49	91	17	35	81.5	228.5
Colorectal	**1673**	50	1	19	61	127	32	0	6	29	90	82	16	46	97	203
Mesothelioma	**57**	42	3	17	34	122	24	1	7	27	84	66	21	41	80	190
Vulval	**49**	59	7	17	61	122	16	0	1	7	54	76	14	31	86	284
Endometrial	**319**	54	1	14	59	168	21	0	1	15	51	75	6	29	83	219
Liver	**53**	28	0	14	39	61	32	1	11	27	73	60	14	36	61	125
Lymphoma	**477**	39	1	14	44	94	33	0	9	31	89	72	16	38	87	186
Ovarian	**275**	39	2	14	52	113	21	0	7	22	51	60	14	36	72	149
Multiple myeloma	**124**	44	0	13.5	31	93	56	5	20.5	62	134	100	22	46	110.5	213
Sarcoma	**72**	45	0	13	56.5	119	45	0	7	40.5	115	90	15	52	111	226
Testicular	**112**	60	2	12	67	184	15	0	1.5	16	30	75	8	24.5	94.5	212
Lung	**1128**	33	0	11	32	85	33	3	14	39	78	66	17	39	79	146
Small Intestine	**38**	43	0	10.5	75	153	26	1	10	32	90	69	12	38.5	128	184
Pancreatic	**268**	26	1	10	31	74	33	0	7	31.5	97	58	9	28.5	74.5	144
Stomach	**187**	45	0	9	38	125	46	0	12	65	134	90	15	49	126	235
Unknown primary	**111**	24	0	8	23	68	33	0	15	36	75	57	14	34	67	129
Brain	**121**	36	1	7	26	92	22	0	4	16	56	58	5	22	61	139
Breast	**2124**	32	1	7	27	77	6	0	0	1	7	38	2	10	31	91
Leukaemia	**228**	32	0	7	30	86	25	0	4	22.5	58	57	6.5	26	68	141
Prostate	**1378**	47	0	7	42	151	31	2	11	26	74	78	9	32	93	209
Gallbladder	**40**	34	0	4.5	15.5	74	41	4.5	20	39	76	75	9	31.5	73.5	304.5
Renal	**207**	26	0	3	21	62	35	1	13	39	108	61	8	30	71	181
Bladder	**602**	22	0	2	14	61	26	0	3	15	53	48	3	14	45	134

Cancers are ordered in descending order of median patient interval. Bootstrap 95% confidence intervals for all estimates are presented in Supplementary Online Material 2.

a Note that centiles occur that are not whole numbers (non-integers) when the centile falls between two observations. In such cases the mean of the two adjacent observations is used.

**Table 2:** Ratio of mean and median patient interval over mean and median primary care interval, by cancer site

**Cancer diagnosis**	**Mean patient interval/Mean primary care interval**	**Median patient interval/Median primary care interval**
Breast	**5.2** (4.0–6.6)	* (*–*)
Melanoma	**4.1** (3.0–5.6)	* (*–*)
Testicular	**4.1** (2.3–7.3)	**8.0** (1.8–*)
Vulval	**3.6** (1.8–9.0)	**17.0** (3.5–*)
Cervical	**3.1** (1.8–5.3)	**5.0** (1.3–39.0)
Endometrial	**2.6** (1.8–3.8)	**14.0** (9.0–*)
Oropharyngeal	**2.4** (1.5–4.0)	**5.5** (1.8–30.5)
Laryngeal	**1.9** (1.1–3.8)	**4.9** (2.4–19.3)
Ovarian	**1.9** (1.4–2.6)	**2.0** (1.1–2.8)
Thyroid	**1.9** (1.1–4.1)	**2.4** (0.9–5.3)
Brain	**1.7** (0.9–3.3)	**1.8** (0.8–8.0)
Mesothelioma	**1.7** (1.0–3.0)	**2.4** (0.7–6.0)
Small intestine	**1.7** (0.8–3.3)	**1.1** (0.2–4.1)
Colorectal	**1.6** (1.4–1.8)	**3.2** (2.6–4.5)
Oesophageal	**1.5** (1.2–1.9)	**4.3** (2.2–11.5)
Prostate	**1.5** (1.3–1.8)	0.6 (0.5–0.8)
Leukaemia	**1.3** (0.8–2.0)	**1.8** (0.6–3.5)
Lymphoma	**1.2** (0.9–1.5)	**1.6** (1.2–2.4)
Lung	1.0 (0.8–1.2)	0.8 (0.6–1.1)
Sarcoma	1.0 (0.5–2.1)	**1.9** (0.4–4.5)
Stomach	1.0 (0.7–1.5)	0.8 (0.3–1.9)
Liver	0.9 (0.4–2.0)	**1.3** (0.5–3.3)
Bladder	0.8 (0.6–1.2)	0.7 (0.3–1.5)
Gallbladder	0.8 (0.3–2.1)	0.2 (0.0–0.7)
Myeloma	0.8 (0.4–1.3)	0.7 (0.3–1.0)
Pancreatic	0.8 (0.6–1.1)	**1.4** (0.8–2.0)
Renal	0.7 (0.5–1.1)	0.2 (0.1–0.5)
Unknown primary	0.7 (0.4–1.3)	0.5 (0.2–1.3)

Values >1.0 are denoted in bold (indicating predominance of the patient over the primary care interval). Bootstrap 95% confidence intervals for all estimates are presented.

*Not estimable because of median primary care interval values of 0.
